# Study of antibiotic consumption using a novel unit among indoor pediatric patients in India

**DOI:** 10.6026/973206300213381

**Published:** 2025-09-30

**Authors:** Aniruddha Ghosh, Nabanita Banerjee, Lopamudra Kirtania, Shah Newaz Ahmed

**Affiliations:** 1 Intern, Maharaja Jitendra Narayan Medical College and Hospital, Coochbehar, West Bengal, India; 2 Department of Community Medicine, Maharaja Jitendra Narayan Medical College and Hospital, Coochbehar, West Bengal, India; 3 Department of Pharmacology, Maharaja Jitendra Narayan Medical College and Hospital, Coochbehar, West Bengal, India

**Keywords:** Pediatric drug audit, antibiotic consumption, defined daily dose

## Abstract

Unlike adult population, the Defined Daily Dose/100 Patient Days (DDD/100 PD) cannot be used as a standard unit in pediatric studies. In this study, we measured antibiotic consumption using standard unit's used- DDD/100 PD and DDD per 1000 Kilogram-Days (DDD/1000 Kg-D) and a novel unit- DDD/100 PD/ Mean Body Weight (DDD/100 PD/MBW). The study cohort was sub-divided into four sub-cohorts (n1=100, n2=100, n3=100, n4=92) with increasing mean body weight (in Kgs) of 6.21±1.46, 9.56±1.00, 13.84±1.59 and 22.94±5.18, respectively. The weight based sub-cohorts sought to explore the most suitable unit for the paediatric population. The Pearson correlation coefficient between DOT and DDD/100 PD, DDD/1000 Kg-D and DDD/100 PD/MBW, were -0.859, 0.981 and 0.975, respectively. Thus, DDD/100 PD/MBW is a novel unit in paediatric drug quantification independent of body weight and age.

## Background:

Drug Utilization Research uses Anatomical Therapeutic Chemical Classification (ATC) classification and Defined Daily Dose (DDD) measurement, as established by WHO, to standardize and compare international, national, and regional, pharmaceutical consumption data [1]. ATC/DDD system ensures comparative analysis of drug utilization, supporting informed decision-making, optimal medication use, and efficient allocation of healthcare resources. System and is accepted universally for drug utilization studies. The DDD is a simple unit for measurement of drug consumption that denotes the average adult maintenance dose per day for a drug's main indication [2]. Based on the ATC/DDD system, the standard unit developed for measurement for drug consumption in adults is Defined Daily Doses per 100 Patient Days (DDD/100 PD). Patient Days (PD) measure hospitalization, where one patient-day equals one patient staying in the hospital for one day. The ATC/DDD system standardizes adult drug utilization monitoring, but its pediatric application requires modifications due to age-specific dosing and pharmacokinetics [3]. In our previous research, we introduced and applied innovative units to quantify drug consumption in pediatric populations: 1. Weight-Specific Defined Daily Dose per 100 Patient Days (WS-DDD/100 PD) 2. Defined Daily Dose per 1000 Kilogram-Days (DDD/1000 Kg-D) [4]. These novel metrics address the limitations of traditional ATC/DDD systems in pediatric settings, providing more accurate drug utilization assessments tailored to children's varying weights and age. These units aim to quantify the consumption of medicines in the pediatric population independent of weight and distribution of a sample. However, these units are computationally laborious and would consume time and resources in large scale studies [5]. In the present work, we have conceived a novel unit of drug consumption, DDD/100 PD/ Mean body weight, where the denominator is the mean body weight in kilograms of all the patients in the cohort. The newer unit is deemed to be effective even in smaller samples and is also free of exhaustive computational burden. Therefore, it is of interest to test the feasibility and effectiveness of the novel unit in quantification of antibiotic consumption across escalating pediatric weight cohorts.

## Materials and Methods:

## Study design:

In this cross-sectional, observational study, medical records of pediatric patients admitted in hospital were accessed through the medical records department of the hospital. Data of patients admitted in a period of two months were collected. The antibiotic consumption for ceftriaxone, a third-generation cephalosporin, was quantified using standard units and the novel unit.

## Standard consumption units:

## Days of therapy (DOT):

DOT is used to measure antibiotic consumption in terms of number of days of antibiotic usage irrespective of dose, route and frequency of administration or spectrum of action. For example, levofloxacin administered by intravenous route in dose of 750 mg once daily for 1 day constitutes one DOT. Likewise, levofloxacin administered by oral route in dose of 500 mg twice daily for 1 day, or once daily for 1 day, both constitute one DOT. The consumption of antibiotics in terms of DOT for a patient on more than one antibiotic is the sum of DOTs of the individual antibiotics. While DOT is limited by its diversity and non-generalisability, its simplicity and widespread use make it an indispensable tool for initial comparative assessments and planning strategic advancements for complex analytics [6].

## DDD/100 patient days (DDD/100 PD):

The quantification of drug consumption for a particular drug in terms of total DDD can be estimated by dividing the multiplication product of number of doses per day, amount of drug per dose and DOT, by the DDD of the drug. Patient-days are the aggregate of the attendance of a patient admitted in a hospital observed at a specific time point in a day. The DDD usage of a drug is expressed commonly in terms of DDD per 100 PD (Figure 1 - see PDF) [7].

## DOT/100 Patient Days (DOT/100 PD):

Conventionally, DOT is expressed as a rate per 100 patient days to enable comparison across centres, regions, and countries. While both DDD/100 PD and DOT/100 PD are used in adult population, only DOT/100 PD can be used in paediatric population, which has representativeness for pattern of drug use but may be misleading for the quantity of drugs consumed. Nevertheless, in absence of a standard appropriate unit for quantification in the child population, DOT and DOT/100 PD is the often-used unit for field operations [6].

## DDD/1000 Kilogram Days (Kg-D):

DDD/1000 Kg-D is a newly conceived standard unit for pediatric drug consumption quantification. In this unit, the weight (in Kilograms) and the length of stay (in days) of the individual patients are multiplied and the products of the two scalers (in Kilogram-Days) are added to serve as the denominator of the unit. The consumption of the antibiotic is expressed in terms of DDD/1000 Kg-D. (Figure 1 - see PDF) [5].

## Novel unit:

A simple and novel unit is proposed in the research work. The novel unit is calculated by dividing the consumption of antibiotic in terms of the standard unit of DDD/100 PD by the mean body weight of the cohort (Figure 1 - see PDF).

## Core concept of the work:

The quantification of antibiotic use in terms of DOT denotes the load of antibiotic usage but fails to quantify the antibiotic consumption in terms of DDD. DDD/100 PD is the standard unit of antibiotic consumption but fails in pediatric population due to weight variability in the pediatric population. Therefore, the linear correlation between DOT and DDD/100 PD in pediatric population is poor. The utility of a novel unit as a standard unit in pediatric population can be assessed using the strength of correlation between DOT and the novel unit. Our work tests the novel unit as a standard unit in pediatric drug audit which is independent of body weight.

## Data collection:

Data was collected from the Medical Records Department. Patient files of the months of July and August, 2023 were accessed. Patients receiving ceftriaxone as one of the antibiotics were identified. The duration of therapy, dose, and frequency of administration of ceftriaxone were recorded. Age, gender, and body weight of the individual patients were obtained. The data was collected and entered in Microsoft Excel sheet and analyzed with statistical tools.

## Data analysis and statistics:

To test the novel unit, ceftriaxone, a third-generation cephalosporin was selected. The consumption of ceftriaxone was calculated in terms of DOT, DDD/100 PD, DDD/1000 Kg-Days and DDD/ 100 PD/ Mean body weight. The strength of correlation between DOT and the standard and novel units were assessed using linear regression. Pearson correlation coefficient was obtained for each correlation. The unit with the maximum value of Pearson correlation coefficient would be adjudged the best unit for standard use.

## Results:

The number of patients included in the study was 392. The number of boys and girls were 250 and 142, respectively. The maximum and minimum age in the study was 12 years and 1 month respectively. The mean age was 4.34±3.65 years. The maximum and minimum body weight was 2.4 and 43 Kg respectively. The mean body weight was 12.94±6.78 Kgs. The maximum and minimum duration of stay in the hospital was 20 days and 1 day, respectively. The mean duration of stay in the hospital was 14.55±8.11 days. The mean age, body weight, duration of stay, total hospital days and mean weight-time products are shown in the Table 1 (see PDF). The data of 392 patients was arranged in order of increasing body weight and the entire cohort (N=392) was divided into four sub-cohorts (n1=100, n2=100, n3=100, n4=92). The mean age of the patients in the four sub-cohorts was 0.87 ± 1.67, 2.57 ± 2.20, 5.19 ± 1.20 and 9.12 ± 2.04 years, respectively. The mean body weight (in Kgs) of the four sub-cohorts was 6.21 ± 1.46, 9.56 ± 1.00, 13.84 ± 1.59 and 22.94 ± 5.18, respectively. The weight-based hierarchy was created to test the suitability of the three units for standard drug quantification in different weight bands of the pediatric population. The mean duration of stay in the hospital in the four sub-cohorts was 3.66±2.52, 2.70±1.76, 2.39 ± 1.76 and 2.27 ± 1.62 days, respectively. The total number of days of hospital stay in the four sub-cohorts was 366, 270, 239 and 209 days, respectively. The cumulative weight-time product of the four sub-cohorts was 2211, 2585, 3245 and 4886 Kg-Days, respectively. The mean weight-time product of the cohort was 32.98 ± 28.24 Kg-Days, and of the four sub-cohorts was 22.11 ± 13.66, 25.85 ± 17.62, 32.45 ± 23.99 and 53.11 ± 40.78 Kg-Days, respectively (Table 1 - see PDF). The consumption of ceftriaxone in the four sub-cohorts (n1, n2, n3, n4) in terms of DOT, DOT/100 PD, DDD/100 PD and DDD/1000 Kg Days was (296, 222, 170, 152), (80.87, 82.22, 71.13, 72.72) (28.46, 39.39, 44.85, 70.33) and (59.88, 49.93, 46.55, 40.86), respectively (Table 2 - see PDF). The consumption of ceftriaxone in the four sub-cohorts in terms of DDD/100 PD/Mean Body Weight was 4.58, 4.12, 3.24 and 3.07, respectively (Table 2 - see PDF). The Pearson correlation coefficient for linear correlation between DOT and DDD/100 PD, DDD/1000 Kg Days and DDD/100 PD/Mean Body Weight, were -0.859, 0.981 and 0.975 (Figure 2 - see PDF), respectively. DDD/100 PD showed negative correlation. DDD/1000 Kg Days and DDD/100 PD/Mean Body Weight showed strong positive correlation.

## Discussion:

Antimicrobial resistance has become a massive public health hazard and it is predicted that by the middle of the century, it will lead to one crore deaths/year globally. An important factor perpetrating the global crisis is the injudicious use of antimicrobials. The micro-organisms that are overexposed to the antimicrobials develop mechanisms to evade and survive the onslaught of antibiotics. Therefore, the quantum of antibiotic usage is an important surveillance tool in antibiotic stewardship programs. It has a direct bearing with the probability of emergence of antibiotic resistance. A significant part of research on antibiotic stewardship is focused on the pattern and quantity of antibiotic use across regions and hospitals. The global consumption and usage of antibiotics are under perpetual vigilance. The objective is to devise measures to retard drug resistance and promote rational prescribing [8]. Worldwide, the ATC/DDD system has been adopted as the standard method of quantifying and comparing antibiotic consumption in adult population. It represents an integrated and universal drug unit for management and control of health systems.

It enables simultaneous utilization, optimization, identification, aggregation and stratification of drug use for development of rational pharmacotherapy and improving clinical outcomes in resource limited settings [9]. Studies have utilized the ATC/DDD system for antimicrobial stewardship intervention programs. In an open-label, cluster-randomized, controlled trial, a multi-faceted computerized antimicrobial stewardship intervention system was randomized among 18 surgical teams (intervention: control=1:1) (2470 patients) for open chest cardiovascular surgery. The consumption of antibiotics was measured in terms of DOT/1000 PD, DDD/1000 PD and length of therapy (LOT)/1000 PD. The consumption of antibiotics, the risk of antibiotic misuse and the expenses on antibiotics was less in the intervention group without any significant difference in the rate of infection or surgical complication between the intervention and the control group [7]. Similarly, many other studies have effectively employed the ATC/DDD system for antimicrobial stewardship and monitoring [10, 11, 12-13]. However, in paediatrics, any DDD-derived method gives very limited information regarding the real number of children receiving medicines and overall DDD results may not be the exact representation for drug consumption comparison. In the pediatric population, where doses are administered per kg body weight for individual patients, the denominator of 100 PD may lead to misleading interpretation. This holds true when we compare drug consumption in two pediatric samples with dissimilar weight distribution. For example, a unit with frequent under dosing but admitting older children may appear to have an average consumption. Owing to such discrepancy, researchers have faced difficulty in enforcing surveillance in antimicrobial stewardship programs in the pediatric population. Zaffagnini *et al.* conducted an electronic App (Firstline.org) based antimicrobial stewardship intervention study in 2021 on antibiotic prescription in a pediatric setting. In this interrupted time series analysis, the historical control, the intervention, and the outcome were assessed/ imparted in the years 2019, 2022 and 2023, respectively. The annual antibiotic consumption was compared before and after the intervention year. In the post-intervention part of the study (n=467), a reduction in antibiotic consumption was observed in terms of defined daily doses per 1000 patient days (-222.13; P<0.001) and days of therapy per 1000 patient days (-452.49; P<0.001). However, the researchers emphatically stated "Antibiotic stewardship interventions in paediatrics are still not standardized regarding methodology, metrics, and outcomes." The results derived meaning because the pre- and post-intervention phases of the study were conducted in the same setting with similar distribution of age and weight of the study cohorts. However, the use of the same units across variable samples differing in mean age and weight may lead to erroneous conclusions [14]. Similar intra-institutional interventional studies for antimicrobial stewardship have successfully achieved desired objective of reduction in antibiotic consumption and rational use of antibiotics [15, 16, 17-18].

However, the studies lacked external validity and was aptly summarized in a narrative exploratory by Meesters *et al.* "while antimicrobial stewardship programs have proven effective in optimizing antibiotic use within inpatient healthcare settings, their implementation in pediatric emergency medicine presents specific challenges." The authors reiterated the necessity of standardized metrics for evaluating antibiotic use in the child population and sought for benchmark initiatives to address this unmet need [19]. Novel units have been proposed in quest of a standard and universally acceptable metric system for pediatric drug quantification. Liem *et al.* had proposed a set of neonatal DDDs for ten commonly used antibiotics in neonates based on an assumed neonatal weight of 2 kg. However, there is a huge variation between neonatal and paediatric weights and neonatal DDD cannot be employed uniformly in all paediatric age groups [20]. In a longitudinal survey of eight years (2004-2011), Elena *et al.* statistically evaluated the increment in antibiotic consumption (in terms of DDD/PD) in Italian Hospitals. No statistically significant temporal association could be observed in the increased consumption of antibiotics (P=0.224). However, it was pointed out that an important limitation of the study was "DDD is intended for adult population and that paediatric age includes children who need different antibiotic doses depending on their body weight" [21]. In a paradigm work by Porta *et al.* the antibiotic consumption was stratified in four standardized weight bands (neonatal, <10, 10-25, >25 Kgs) and inter-centric comparison was attempted, albeit, with limited clinical usefulness [3].

The physiological changes determining pharmacokinetic variation in the neonatal-pediatric adolescent transition phase is reflected in the weight categories [22]. Alonso *et al.* proposed a pediatric DDD for a cohort by multiplying the recommended dose in mg/Kg with the weight of the median age of the cohort [23]. The proposal was repeated by the researchers in a separate study which was conducted in two phases. In the phase I done in 10 Spanish hospitals, the pediatric DDD was calculated theoretically for 44 antimicrobials by multiplying the weight (kg) of the median age by sex of the 50th percentile (as per WHO weight for age chart) with the antimicrobial dose agreed upon by the expert panel (mg/kg). The theoretical DDDs were validated in the Phase II of the study. There was disagreement ("heterogenous distribution") between the DDD of the two phases and the authors stated the reason thereof as "if we analyze the demographic data of Phase I, we can observe that they are lower than those of Phase 2, 4.43 vs. 6.64 years and 17.08 vs. 25.70 kg. Therefore, the doses used in the validation phase will be generally higher, and this would explain why antibiotic consumption, expressed in DDD, is also higher." Nevertheless, the authors contended that ages were "relatively similar" and "consistent" and extrapolated the results to "the general pediatric population" [24]. Earlier studies attempted multiple measures of quantification of antibiotic in pediatric population with limited success. Some of the methods were simple but inconsistent like number of vials per day per patient per day. Improvements were suggested in the form of age-adjusted daily use of vials. A better unit referred to as the recommended daily dose (RDD) was suggested with reference to the 50th percentile weight for age for the cohort under consideration. However, all such devised methods of antibiotic consumption in pediatric population could not meet standards for comparability across heterogenous study groups [25]. Antibiotics are most commonly prescribed group of drugs in the pediatric age group. In a cross-sectional study conducted in the Pediatrics department of a tertiary centre in West Bengal, it was found that 39.25% of the patients were receiving antibiotics for infections [26]. The quantification of antibiotic consumption, therefore, holds immense importance in antimicrobial stewardship in this special population. In our previous studies, we had conducted two pilot studies to quantify medicine consumption in this special population. In the first study, we had developed a novel metric system based on quantifying drug consumption in terms of DDD/100 PD in discrete weight categories. Though a very robust unit for comparison and quantification, it involves segregating the sample into multiple discrete weight based sub-samples, and then quantifying and comparing drug consumption across similar sub-samples of other audits [4]. In the second study, another novel unit, namely DDD/1000 Kg-Days, wherein the denominator is the sum-total of the products of body weight and length of stay of the individual patients, was used [5]. Both the units were able to quantify drug consumption in the pediatric population independent of weight and age of the study groups and have the potential to serve as a standard unit for comparison across local, national, and trans-continental geographical regions. However, both the units are computation intensive and a simple yet effective unit was desirable. In the newly proposed unit, the antibiotic consumption in DDD/100 PD (the standard unit in adult population) when divided by the mean body weight of the respective cohort, becomes independent of weight and age of the cohort, enabling comparability for stewardship studies. The proposition is strongly substantiated by the strong correlation (0.975) between DOT and the novel unit- DDD/100 PD/mean body weight. Though mathematically weaker than the correlation between DOT and DDD/1000 Kg-days (0.981), the difference is practically negligible, and computationally liberal and free from cumbersomeness. The new unit, in our parlance is a potential game changer and a paradigm leap in conventional drug quantification studies in pediatrics.

## Conclusion:

DDD/100 PD/ mean body weight is a simple unit with potential prowess of becoming a standard unit in pediatric drug audit immune to confounding factors. The present exploratory study merits future studies on a larger scale to escalate the use of the unit to global standards.
